# Community health nurses’ learning needs in relation to the Canadian community health nursing standards of practice: results from a Canadian survey

**DOI:** 10.1186/1472-6955-13-31

**Published:** 2014-10-21

**Authors:** Ruta K Valaitis, Ruth Schofield, Noori Akhtar-Danesh, Andrea Baumann, Ruth Martin-Misener, Jane Underwood, Sandra Isaacs

**Affiliations:** 1 School of Nursing, McMaster University, 1280 Main Street West, Hamilton ON L8S 4K1, Canada; 2 School of Nursing, Dalhousie University, 6299 South St, Halifax NS B3H 3J5, Canada

**Keywords:** Community health nursing, Learning needs, Standards of practice, Professional education, Continuing education, Undergraduate education, Nursing education

## Abstract

**Background:**

Canadian Community health nurses (CHNs) work in diverse urban, rural, and remote settings such as: public health units/departments, home health, community health facilities, family practices, and other community-based settings. Research into specific learning needs of practicing CHNs is sparsely reported. This paper examines Canadian CHNs learning needs in relation to the 2008 Canadian Community Health Nursing Standards of Practice (CCHN Standards). It answers: What are the learning needs of CHNs in Canada in relation to the CCHN Standards? What are differences in CHNs’ learning needs by: province and territory in Canada, work setting (home health, public health and other community health settings) and years of nursing practice?

**Methods:**

Between late 2008 and early 2009 a national survey was conducted to identify learning needs of CHNs based on the CCHN Standards using a validated tool.

**Results:**

Results indicated that CHNs had learning needs on 25 of 88 items (28.4%), suggesting CHNs have confidence in most CCHN Standards. Three items had the highest learning needs with mean scores > 0.60: two related to epidemiology (means 0.62 and 0.75); and one to informatics (application of information and communication technology) (mean = 0.73). Public health nurses had a greater need to know about “…evaluating population health promotion programs systematically” compared to home health nurses (mean 0.66 vs. 0.39, p <0.010). Nurses with under two years experience had a greater need to learn “… advocating for healthy public policy…” than their more experienced peers (p = 0.0029). Also, NPs had a greater need to learn about “…using community development principles when engaging the individual/community in a consultative process” compared to RNs (p = 0.05). Many nurses were unsure if they applied foundational theoretical frameworks (i.e., the Ottawa Charter of Health Promotion, the Jakarta Declaration, and the Population Health Promotion Model) in practice.

**Conclusions:**

CHN educators and practice leaders need to consider these results in determining where to strengthen content in graduate and undergraduate nursing programs, as well as professional development programs. For practicing CHNs educational content should be tailored based on learner’s years of experience in the community and their employment sector.

## Background

This paper examines Canadian community health nurses’ (CHNs) learning needs in relation to the 2008 Canadian Community Health Nursing Standards of Practice (CCHN Standards) [1]. CHNs in Canada work in diverse urban, rural, and remote community settings such as: public health units/departments, home health, community health facilities, family practices, and other community based settings (e.g., faith organizations, shelters, schools) [2]. Canadian CHNs’ role is to “promote, protect, and preserve the health of individuals, families, groups, communities, and populations in the settings where they live, work, learn, worship and play in an ongoing and/or episodic process”, [3] (pg 4). The 2008 CCHN Standards define practice expectations of CHNs working in clinical practice, education, administration and research. They provide a benchmark that new CHNs are expected to achieve within two years of experience. CHNs “are expected to know and use the following five standards of practice: 1. promoting health which includes: a) health promotion, b) prevention and health protection, and c) health maintenance, restoration and palliation; 2. building individual and community capacity; 3. building relationships; 4. facilitating access and equity; and 5. demonstrating professional responsibility and accountability” (pg. 10). Relating CHNs’ learning needs to these standards informs graduate and undergraduate nursing curricula, professional development, and performance monitoring.

The CCHN Standards are widely recognized by community health nursing educators however, one study indicated that they were rarely used in guiding course objectives or framing evaluations in Canadian undergraduate nursing programs [4]. A Canadian CHN textbook that is heavily used in Canadian nursing schools explores the CHNC Standards in an introductory chapter [5]. A subcommittee of the Canadian Association of Schools of Nursing which is responsible for accrediting all Canadian schools of nursing, released guidelines for quality community health placements. The first essential guideline states that: “Faculty advisor/clinical instructor has knowledge of the Canadian Community Health Nursing Standards of Practice, primary health care principles, public health sciences and nursing science” [6,7].

Research into the specific learning needs of practicing CHNs is sparsely reported in the literature. This is complicated by the diversity of role definitions and settings in which CHNs work. Much like CHNs in other Canadian provinces, Ontario CHNs work in a variety of different settings [8]. Armstrong-Stassen and Cameron [9] acknowledged differences among Ontario CHNs’ work-related concerns, job satisfaction, and factors influencing decisions to remain in community health nursing depending on their workplace setting. These authors concluded that it is important to treat CHNs as a heterogeneous group and policies and practices should be tailored to address concerns specific to nurses’ workplace. Given this, it seems prudent to assess learning needs by CHN workplace. A recent Registered Nurses Association of Ontario report identified that registered nurses (RNs) and registered practical nurses (RPNs) “represent a grossly under-utilized resource in Ontario’s primary care system, and these nurses are waiting and eager to take on expanded roles” [10] (p.16). The authors recommend that primary care nurses self-assess their educational needs and engage in educational programs to meet role requirements. Another Ontario study exploring strategies to retain home care nurses indicated that organizational supports were required to maintain a stable workforce including provision of learning supports which were lacking in home care workplace settings [11]. Research has also showed that Ontario CHNs perceived what they were doing on the job was important and having adequate time and training to perform them were important factors influencing their decision to remain in community health nursing [9]. Finally, Underwood and colleagues [12] identified that only 45% of Canadian CHNs in practice felt they had “the learning opportunities they needed, including adequate time, money and access to learning resources” (pg. I-6).

Under-graduate students, rely on community placements to gain knowledge and experience in community nursing [13,14]. Yet, educators identified that finding appropriate placements was dependent upon availability of CHNs as preceptors as well as qualified faculty [13](p.15). A Canadian environmental scan revealed that preceptors felt their knowledge and application of primary health care principles and the CCHN Standards were insufficient to prepare nurses for community practice [15]. However, this may be changing due to the incorporation of the CHNC Standards by some home care and public health employers in performance appraisals. The quality of Canadian education programs in community health nursing can have an impact on the knowledge, skills and abilities of new graduates.

CHNs are older on average than other nurses. In 2007, 28% of these RNs were over 55 [16] (p.2). With much of the workforce out of school for as many as 20 to 30 years, knowledge needed by CHNs to implement current practice standards requires greater attention. Policy statements acknowledge a shift toward health promotion, disease prevention and community health care in response to an aging population and soaring costs of hospital care [16,17]. This accentuates the need to investigate learning needs of CHN nurses in the midst of health systems change.

Since 2006, the Canadian Nurses Association (CNA) has supported a voluntary CHN certification program based on the most current CCHN Standards [18]. As of 2013, there were 840 certified nurses in community health nursing in Canada [19]. Given that more than 46,000 RNs work in community health in Canada [16], the lack of mandatory certification for practicing CHNs creates a need for other means to determine their competencies and understand their learning needs.

To address this need, a research study was conducted involving two phases. The first phase involved the development and testing of a tool to measure CHNs’ learning needs based on the CCHN Standards. Testing was conducted in 2 provinces, Ontario and Nova Scotia. The development phase was described in detail elsewhere [20]. Phase II involved the use of the revised validated tool in a national survey with CHNs across Canada. The research questions were:

1. What are the learning needs of CHNs in Canada in relation to the CCHN Standards?

2. What are differences in CHNs’ learning needs by: province and territory in Canada, work setting (home health, public health and other community health settings) and years of practice as a nurse?

This paper focuses on the results of Phase II and answers the above research questions which can inform educators and practice leaders regarding areas for capacity development of CHNs. In this article we only report the results on the learning needs items with a mean score of 0.5 or larger.

## Methods

The questionnaire (Additional file 1) was developed by research team members with expertise in community health nursing and the CCHN Standards. The instrument consisted of items representing nursing activity statements based on the CCHN Standards published in 2008. Items were listed under five core CCHN Standards. The stem question, “**I perform the stated activity**” with a corresponding response scale of never, rarely, sometimes, frequently and always, was meant to obtain information about the frequency of performance per item listed. Respondents could alternatively indicate ‘not applicable’ or ‘unsure’. The stem question, “**I need more education related to this activity**” with a 5-point Likert scale based on degree of agreement (completely agree, generally agree, neither agree or disagree, generally disagree and completely disagree), measured continuing education needs per item. Instrument pretesting established face validity and test-retest reliability of the survey items (activity statements) for both the activity performed and learning need scales based on 329 responses from CHNs in Phase I. Test-retest reliability measured r = .890 with p < 0.01for the learning need scale and r = .889 with p < 0.01 for the activity performed scale. Responses were also used to further refine the instrument, dropping items that were highly correlated (r ≥ =0.80) with another of similar meaning on the learning need scale; the higher conceptual level statement was generally retained [20]. There were 88 items retained in the revised questionnaire used in Phase II compared to 138 items in the Phase I pilot test questionnaire. French translation, including reverse translation, was conducted with the assistance of a linguist to ensure clarity of language in both phases. Face validity testing was also conducted by bilingual nurses on the French version of the Phase II questionnaire.

Phase II of the study involved a random sampling of CHNs from every province and territory in Canada. Recruitment occurred between late 2008 and 2009. Participants’ names were obtained from the nursing regulatory associations or colleges from each province/territory. CHNs’ met the inclusion criteria as shown in Table 1.

**Table 1:** Inclusion criteria for study selection

**Inclusion criteria**	
1. Gave permission to participate in research on their regulatory body’s registration forms	
2. Worked for any of the following employers:	
○ Mental health centre	○ Educational institution
○ Community health centre	○ Association/government
○ Community health agency	○ Self-employed/independent practice
○ Community nursing clinic	○ Extra-mural program
○ Home care agency	○ Parish nursing
○ Public health unit/department	○ Outpost/nursing station
○ Private nursing agency	○ Indian Reserve
○ Visiting nursing agency	○ First Nations and Inuit Health Branch
○ Nursing station (outpost or clinic)	○ Armed forces
○ Physician’s office/family practice unit	○ Addiction centre
○ Business/industry/occupational health	○ Other community
3. Were French or English speaking	
4. Credentialed as RNs, primary health care NPs, RN (extended class (EC), graduate nurses, and psychiatric nurses	
And	
5. Were employed full, part time, or on a casual basis	

To estimate the mean of learning needs with a power of 0.95 and a maximum effect size of 0.5 from each provincial/territorial jurisdiction, a sample size of 16 nurses was needed [21]. Based on a response rate of 40% (supported by Phase I) the sample size increased to 40. To conduct statistical comparisons by subgroups, the sample size was multiplied by 4 and increased to 160 per jurisdiction. To further increase the power of the study allowing for future factor analysis, we anticipated randomly selecting 350 nurses from the provinces and territories of Nova Scotia, Newfoundland and Labrador, New Brunswick, Quebec, Ontario, Manitoba, Saskatchewan, Alberta, and British Columbia; 150 nurses from PEI; 100 from Yukon, and 50 each from Northwest Territories and Nunavut; these estimates were proportional to the population size of each jurisdiction. The final sample size calculation was 3500 CHNs across Canada. Therefore, based on 40% response rate the final expected sample size is 1400 which is large enough for all the expected estimations and statistical testing. Then, the required number of CHN’s for each province/jurisdiction was randomly selected using a randomization table from lists of CHN’s provided by each respective nursing regulator.

The protocol followed a modified Dillman approach [22] involving a pre-notice postcard (day 1), a mailed questionnaire (day 4), a thank you/reminder postcard (day 11), and a second questionnaire mailing (day 24) to remaining non-respondents. All mailed questionnaires included a stamped return envelope. The study was approved by the Hamilton Health Sciences/McMaster University Faculty of Health Sciences Research Ethics Board, the Health Sciences Human Research Ethics Board of Dalhousie University, and the Aurora Research Institute, Aurora College, Inuvik, NT. All other jurisdictions accepted these ethics approvals.

Data was analyzed using Stata SE/11.1 [23]. Descriptive statistics were generated on responses for learning needs items including frequency counts, measures of central tendency, and measures of dispersion. Items were considered high learning needs if mean scores for ‘learning needed’ responses were equal to or greater than 0.5 (response scale of -2 to +2). Respondents were grouped according to employment sector/setting [public health, home health, and other including primary care, educational institutions, occupational health, etc.], title in nursing [RN, RN (EC), nurse practitioner (NP)], province/territory of employment, and number of years in practice.

Differences among subgroup mean scores were examined using one-way ANOVA followed by a Tukey HSD pairwise comparison for items identified as the top 10 learning needs (highest mean scores); this selection excluded items referencing three theoretical frameworks: the Ottawa Charter, the Jakarta Declaration, and the Population Health Promotion Model. Items referencing these three frameworks were treated separately since they were conceptually different and reflected knowledge rather that activity statements. We also instructed respondents that if they were unfamiliar with the content, they could respond ‘unsure’ to the ‘activity performed’ question, and indicate their learning need against the item.

## Results

Of 3422 CHNs sampled, 1677 responded, resulting in a 49% response rate. The total number of usable questionnaires was 1344 or 39.3% of the total population surveyed. Response rates by province/territory ranged from 19% (Northwest Territories) to 74% (Nunavut). Study participants were 94.5% female. Their mean age was 49.2 years (SD = 9.7). The majority of nurses were RNs (82.2%), while 15.5% were either RN (EC) or NPs. The highest level of education attained in nursing was a Doctorate (0.1%), Masters degree (4.6%), however, the majority of nurses held a diploma (39.5%) or Baccalaureate degree (53.5%) in nursing. A small group identified having a certificate (2.3%) as their highest level however we were unable to determine what types of certificates these were. Almost half (47.5%) were employed in nursing for over 25 years, while 26.4% worked for 16-25 years. The largest group (21.4%) worked specifically in the community for 6-10 years, while 12.8% of nurses did so for more than 25 years. Most nurses indicated that they worked in a public health unit/department (22.7%) or in a public or private home health agency (23.5%). Other workplaces included primary care settings such as a physician’s office or a community health centre (18.5%), community health agency (9.9%), and mental health centres (4.7%). The most common positions based on primary employers were public health nurses (21.2%) followed by home health nurses (14.1%).

Tables 2 and 3 present mean scores for items with learning need scores greater than or equal to 0.5 (i.e. “I need more education related to this activity”) listed under each of the five Standards. Table 2 lists all items other than those referencing the three theoretical frameworks. Twenty-five of a possible 88 items (28.4%) scored at or above the cut off mean of 0.5. 

**Table 2:** CHN identified learning needs (Learning need mean scores ≥0.5) compared to activity performance

**Activity statements (Items) for each CCN standard**	**Learning need**^**1**^	**Activity performed**^**2**^
	**Mean (SD) n**	**Mean (SD) n**
**Standard 1a: Health Promotion**		
I use research findings.*	0.59 (0.95) 1249	3.52 (0.91) 1303
I address root causes of illness and disease.	0.50 (0.99) 1229	3.88 (0.95) 1265
I use social marketing strategies to shift social norms.	0.50 (1.00) 1192	2.40 (1.05) 1112
In partnership with stakeholders, I evaluate population health promotion programs systematically.*	0.53 (1.02) 1172	2.51 (1.16) 1066
**Standard 1b: Prevention & Health Protection**		
In a variety of contexts, including home, neighbourhood, workplace, school and street, I utilize harm reduction principals to reduce risk factors.	0.50 (0.98) 1206	3.91 (0.97) 1224
I engage in collaborative intersectoral partnerships to address prevention issues.	0.50 (0.97) 1194	3.27 (1.12) 1081
I evaluate collaborative practice (i.e., personal, team, and/or intersectoral) in achieving individual/community health outcomes.	0.50 (0.95) 1208	3.49 (1.08) 1228
I apply epidemiological principles in using strategies (such as, a) screening, b) surveillance, c) communicable disease response, d) outbreak management, and e) education).*	0.62 (1.02)1202	3.66 (1.10) 1114
**Standard 1c: Health Maintenance, Restoration & Palliation**		
I recognize trends in epidemiological data.*	0.75 (0.99) 1197	3.29 (1.03) 1100
I facilitate maintenance of health in response to significant emergencies that negatively impact upon the health of clients.	0.50 (0.97) 1196	3.80 (1.02) 1131
**Standard 2: Building Individual and Community Capacity**		
I use community development principles when I engage the individual/community in a consultative process.*	0.58 (0.98) 1188	3.71 (1.01) 1082
I use community development principles when I use empowering strategies (such as mutual goal setting, visioning, and facilitation).	0.50 (0.99) 1214	3.74 (0.98) 1185
I use community development principles when I use facilitation skills to support group development.	0.52 (1.00) 1182	3.41 (1.12) 1052
I use community development principles when I assist the group/community to marshal available resources to support taking action on their health issues.*	0.52 (0.98) 1175	3.30 (1.05) 1040
I use a comprehensive mix of community/population based strategies (such as coalition building, intersectoral partnerships, and networking) to address issues of concern to groups/populations.*	0.55 (1.01) 1167	3.02 (1.14) 992
I use principles of social justice to support those who are unable to take action for themselves.*	0.57 (0.99) 1215	3.54 (1.13) 1167
**Standard 3: Building Relationships**		
I am aware of culturally relevant communication in building relationships.	0.50 (1.02) 1246	4.29 (0.77) 1305
**Standard 4: Facilitating Access and Equity**		
I provide culturally relevant care in diverse communities.	0.50 (1.00) 1211	3.72 (1.03) 1192
To address service accessibility issues, I take action, based on evidence, with individuals/communities at the federal level.	0.50 (1.04) 1152	1.91 (1.15) 1000
I advocate for healthy public policy, by participating in legislative and policymaking activities that influence health determinants.*	0.54 (1.01) 1183	2.27 (1.14) 1094
**Standard 5: Demonstrating Professional Responsibility and Accountability**		
I use nursing informatics (i.e., information and communication technology) which includes generation, management, and processing of relevant data to support nursing practice.*	0.73 (0.96) 1250	3.67 (1.06) 1255
I use available resources to systematically evaluate community health nursing practice (e.g., availability, acceptability, quality, efficiency, and effectiveness).	0.53 (0.95) 1208	3.40 (1.05) 1188

^1^Learning need: “I need education related to this activity” Score range: -2 to +2.

^2^Activity Performed: “I perform the Stated Activity” Score range: 1 (Never) to 5 (Always).

*10 items with the highest learning need mean scores.

**Table 3:** CHN learning needs with reference to three theoretical frameworks

	**Learning need**	**Activity performed**
**Activity statements related to theoretical frameworks**	**Mean (SD) n**	**Mean (SD) n**
I facilitate planned change through applying the *Population Health Promotion Model.*	1.09 (0.98) 1225	3.16 (1.31) 534
I implement health promotion strategies based on the *Ottawa Charter.*	1.17 (0.97) 1215	3.43 (1.21) 389
I facilitate action in support of the five priorities of the *Jakarta Declaration.*	1.31 (0.90) 1223	2.64 (1.40) 230

As shown in Table 2, items under all five standards were identified as high learning needs (Standard 1 having identified items under all 3 sub-standards). Three items stood out above others as learning needs with mean scores greater than 0.60: two related to epidemiology (means 0.62 and 0.75); and the third to informatics or the application of information and communication technology (mean = 0.73).

As shown in Table 2, twelve items identified as high learning needs (mean ≥0.5) were also reported to be activities performed more frequently on average (mean >3.5). Noteworthy were learning needs for culturally relevant communication and culturally relevant care, the application of epidemiological, harm reduction, community development and social justice principles, health maintenance in response to emergencies, and the use of nursing informatics. Other items were identified as high learning needs, although performing these activities was low, on average (<2.5). They included: social marketing to shift social norms; taking action at the federal level to address service accessibility issues, and participating in legislative and policy making.

Results specific to the three theoretical frameworks are shown in Table 3. When asked about performance on these items, a large proportion of respondents were ‘unsure’ indicating a potential lack of familiarity with the concept. Of 1344 survey participants, 49.0% (658), 57.5% (773) and 68.4% (919) indicated that they were *unsure* if they performed activities relating to the Population Health Promotion Model [24], the Ottawa Charter of Health Promotion [25], or the Jakarta Declaration [26] respectively. The mean scores of respondents who recognized the frameworks for activity performed (did not choose “unsure”) were comparable to other types of activities. Overall, however, learning need mean scores for these items were higher than all others, that is, greater than 1.0 compared to 0.75, the highest learning need score among the top 10 in Table 2.

### Comparison of means among and between groups

Tables 4 and 5 present results of the ANOVA analysis demonstrating differences in means among and between groups on the top 10 learning needs identified in Table 2. Only results with statistically significant findings (p ≤0.05) are displayed in Table 4. Learning needs of CHNs were significantly different on one or two items depending on: their employment setting, years in nursing, and professional title (Table 4). Public health nurses had a greater need to know about ‘…evaluating population health promotion programs systematically’ compared to home health nurses. Nurses with less than two years experience had a greater need to learn about ‘… advocating for healthy public policy, by participating in legislative and policymaking activities that influence health determinants’ than their more experienced peers. NPs had a greater need to learn about ‘…using community development principles when engaging the individual/community in a consultative process’ compared to RNs. When considering province/territory of employment, however, learning needs were considerably different for CHNs on 8 of top 10 items (Table 5). Overall, CHNs from PEI and the Territories appeared to have the strongest learning needs within the top 10 activities considered, with the exception of CHNs in Newfoundland (NL) who also had a greater learning need regarding ‘advocacy for healthy public policy’.

**Table 4:** Mean (SD) of CHN learning needs by employment sector, years in nursing, and nursing title

**Employment sector**								
	**Public health**		**Home health**		**Other**			**F (df1, df2), P-value**
In partnership with stakeholders, I evaluate population health promotion programs systematically	**0.66 (0.95)**		0.39 (1.08)		0.54 (1.01)			6.03 (2, 1156), p = 0.0025*
**Number of Years in Nursing**								
	**Under 2 years**	**2-5 years**	**6-10 years**	**11-15 years**	**16-20 years**	**21-25 years**	**25 + years**	
I advocate for: healthy public policy, by participating in legislative and policymaking activities that influence health determinants.	**1.08 (0.76)**	0.67 (1.02)	0.84 (0.81)	0.53 (1.07)	0.58 (1.03)	0.63 (0.92)	0.44 (1.03)	3.33 (6, 1163), p = 0.0029*
**Title in Nursing - RN, RN Extended Class, NP**								
	**RN**		**RN Extended**		**NP)**			
I use community development principles when I engage the individual/community in a consultative process.	0.56 (0.99)		0.60 (0.91)		**0.91 (0.76)**			2.89 (2, 1162), p = 0.056*

*F value significant at p < 0.05. **Bolded** values indicate a higher learning need compared to at least one other group as identified through Tukey HSD comparisons of means.

**Table 5:** Differences in CHN learning needs by province/territory (means, standard deviations and F values)

**Top 10 learning needs (mean >0.5)**	**BC**	**AB**	**SK**	**MB**	**ON**	**QC**	**NB**	**PEI**	**NS**	**NL**	**TERR**	**F**
I recognize trends in epidemiology data.	0.71 (1.07)	0.70 (1.06)	0.74 (1.00)	0.69 (1.09)	0.79 (0.95)	0.49 (1.15)	0.78 (0.88)	**1.05 (0.81)**	0.78 (0.93)	0.88 (0.86)	**0.97 (0.89**)	F = 1.83 (df 10, 1186) p = 0.0517*
I use nursing informatics (i.e., information and communication technology) which includes generation, management, and processing of relevant data to support nursing practice.	0.58 (1.01)	0.64 (0.93)	0.65 (1.03)	0.65 (0.96)	0.61 (1.02)	0.84 (0.96)	0.94 (0.84)	**1.05 (0.82)**	0.72 (0.95)	0.81 (0.95)	0.97 (0.78)	F = 2.47 (df 10, 1239) p = 0.0063*
I apply epidemiological principles in using strategies such as, a) screening, b) surveillance, c) communicable disease response, d) outbreak management and e) education.	0.60 (1.07)	0.51 (1.04)	0.42 (1.09)	0.50 (1.11)	0.53 (1.04)	0.71 (0.96)	0.70 (1.10)	0.88 (0.84)	0.65 (0.94)	0.79 (0.87)	**0.92 (0.81)**	F = 2.31 (df 10, 1191) p = 0.0108*
I use research findings.	0.46(1.00)	0.57 (0.88)	0.55 (1.01)	0.58 (0.91)	0.44 (1.01)	0.69 (0.97)	0.73 (0.92)	0.53 (0.88)	0.57 (0.89)	0.70 (0.95)	0.81 (0.93)	F = 1.50 (df 10, 1238) p = 0.1348
I use community development principles when I engage the individual/community in a consultative process.	0.67(1.03)	0.51 (0.99)	0.34 (1.05)	0.69 (0.99)	0.52 (0.91)	0.52 (0.85)	0.58 (1.02)	**0.90 (0.86**)	0.51 (1.02)	0.61 (0.92)	**0.93 (0.83)**	F = 2.68 (df 10, 1177) p = 0.0030*
I use principles of social justice to support those who are unable to take action for themselves.	0.67 (0.95)	0.52 (1.03)	0.46 (0.98)	0.47 (0.96)	0.56 (0.95)	0.50 (1.02)	0.59 (1.08)	0.72 (0.88)	0.52 (1.04)	0.66 (0.97)	0.81 (0.86)	F = 1.18 (10, 1204) p = 0.2988
I use a comprehensive mix of community/population based strategies (such as coalition building, intersectoral partnerships, and networking) to address issues of concern to groups/populations.	0.56 (1.02)	0.46 (1.03)	0.41 (1.02)	0.66 (1.02)	0.49 (0.98)	0.39 (1.02)	0.64 (1.02)	0.65 (1.00)	0.49 (1.02)	0.66 (0.97)	**0.89 (0.90)**	F = 1.94 (10, 1156) p = 0.0365*
I advocate for healthy public policy, by participating in legislative and policymaking activities that influence health determinants.	0.58 (1.02)	0.41 (1.03)	0.56 (0.97)	0.62 (1.00)	0.57 (0.96)	0.18 (1.02)	0.53 (1.04)	**0.79 (0.97)**	0.57 (1.02)	**0.71 (0.99)**	**0.70 (0.94)**	F = 2.57 (df 10, 1172) p = 0.0044*
In partnership with stakeholders, I evaluate population health promotion programs systematically.	0.45 (0.97)	0.43 (1.10)	0.53 (1.06)	0.52 (1.07)	0.32 (1.06)	0.51 (0.92)	0.58 (1.01)	**0.86 (0.87)**	0.39 (1.04)	0.73 (0.93)	**0.97 (0.84)**	F = 3.17 (df 10, 1161) p = 0.0005*
I use community development principles when I: d) assist the group/community to marshal available resources to support taking action on their health issues.	0.40 (1.04)	0.47 (1.01)	0.39 (1.01)	0.57 (1.04)	0.41 (0.95)	0.57 (0.85)	0.57 (1.01)	**0.86 (0.84)**	0.51 (1.02)	0.51 (0.92)	**0.86 (0.86)**	F = 2.055 (df 2, 1164) p = 0.0254*

*F value significant at p < 0.05. **Bolded** values indicate a higher learning need compared to at least one other group as identified through Tukey HSD pairwise comparisons of means, where HSD Test value > critical value.

## Discussion

Our findings point out that 80% of the items in our survey were not identified as a learning need, thereby demonstrating that CHNs are confident in most of the Standards. It is important to note that although nurses identified a need for more education in 28.4% of the items, this does not imply that they know nothing about these topics. It is commendable that CHNs recognize areas of practice for improvement and identify evolving knowledge and evidence about which they want to learn more. Results should be used in practice to guide the development of topics for professional development for CHNs.

There was an identified need to know more about applied epidemiology including infectious disease surveillance and response. The study was conducted around the time of the H1N1 outbreak in 2009, however, CHNs would have experienced the impact on their working lives of the SARS outbreak in 2002/2003 and for some, that of the Walkerton e-coli/campylobactor outbreak in 2000 [27]. The demands of these experiences could have accentuated the need for preparedness in the field of epidemiology to anticipate and respond to similar crises in future. Subsequent on-line learning models on applied epidemiology have been developed by the Public Health Agency of Canada to increase epidemiological capacities within Canada’s public health system; however, these modules were not made available to CHNs working outside of public health. Applied epidemiology should be embedded in nursing education programs and made more accessible to nurses beyond the public health sector. Furthermore, in 2014, Canadian Schools of nursing were expected to support new entry to practice public health nurse competencies for all undergraduates [28]. There is a specific competency on “population and community health assessment and analysis” with an indicator related to recognizing trends and patterns in epidemiology.

On observation, other learning needs of considerable concern to the authors were items identified not only as high learning needs, but also as frequently performed activities, indicating an immediate need to address them. These included - culturally relevant communication and culturally relevant care, harm reduction, community development, social justice principles, health maintenance in response to emergencies, and the use of nursing informatics. These learning needs have been reported by others as important areas for development for Canadian CHNs, particularly social justice [14], technology [29-31], and cultural competence [32].

Employers should take an evidence-based approach to planning continuing education, understand the learning needs of their staff, and provide them with time and resources to support skills development to reinforce their current roles. Tremblay and colleagues report on the use of reflexivity to support professional development in health promotion and practice [33]. With reliance and emphasis on current practitioners to mentor undergraduates in community health practice having knowledge of the CHN Standards [6], as well as to mentor their peers [10,11,34,35], and with the gap in existing curricula to prepare CHNs [15], the profession needs to seriously consider how the practice of community health nursing is likely to progress in Canada. This needs to be of concern to nursing educators, employers and policy makers in the field, particularly given the current dynamics of our health systems which intend to move health ‘care’ into community [36]. From a fiscal perspective, this is also important given the emphasis of health promotion and disease prevention to alleviate the costs of curative, hospital-based care.

Other items not frequently performed by CHNs that scored high as learning needs included: social marketing to shift social norms, taking action at the federal level to address service accessibility issues, and participating in legislative and policy making activities. Although these activities are more likely to be performed by CHNs in specialized areas of advanced practice, it is unclear why respondents generally expressed a desire to learn these skills. This may reflect changing expectations of employers. Kulig and colleagues see rural nurses as key players to influence policy in the communities they serve and argue that nurses require educational preparation in policy development, implementation and evaluation [37]. Qualitative research involving CHNs would be helpful to interpret these findings and highlights an area for further study.

The results concerning the three theoretical frameworks – the Ottawa Charter [25], the Jakarta Declaration [26], and the Population Health Promotion Model [24] – suggest that CHNs may be unfamiliar with them. With the large proportion of CHNs having graduated prior to their publication in the late 80′s and 90′s, many would not have had exposure to these foundational theories in their undergraduate preparation. Under current CCHN Standards, understanding these frameworks is pivotal to community health nursing practice. Therefore, practitioners are encouraged to be continuous learners, practicing from a theoretical base to ensure a depth of practice that is integrated with a professional identity and value set. Both CHNs and their employers need to have knowledge of the principles embedded in these frameworks if CHNs are to carry out the full scope of their practice potential. This underscores the need to strengthen knowledge of these relevant theories in undergraduate educational programs as well as to embed them as key underlying concepts in professional development programs.

Only one significant difference was found with respect to learning needs by work setting. Public health nurses had a stronger learning need compared to home health nurses in being able to evaluate population health promotion programs systematically in partnership with stakeholders. This likely reflects different role expectations of public health nurses compared to other CHNs [38-41]. Differences in learning needs were also found with respect to years in nursing practice and nursing title. Importantly, when comparing responses from the different provinces and territories, CHNs differed on eight of the top ten learning needs. How this reflects differences in core competencies supported in undergraduate preparation, professional development opportunities following graduation, or the role expectations of different provinces and territories reinforcing different learning needs requires more research.

It is important to point out some limitations in this study. Researcher bias may have been introduced when respondents were directed to identify theoretical framework items as learning needs if they responded ‘unsure’ to the ‘activity performed’ question. A CHN who is not expected to conduct a particular activity in her workplace might not identify it as a learning need. This is why the researchers were interested in knowing if respondents performed the activities. However, it is possible that activities were not being performed due to a lack of knowledge about the item. In addition, 60% of CHNs surveyed did not respond to the questionnaire or else their questionnaires were not useable, introducing a potential response bias in the results. However, a comparison of respondent descriptors to CHN workforce statistics [8] indicates representativeness among study participants. In addition, the survey is a self-report of CHNs assessment of their use and knowledge of the CCHN Standards. It is not intended to provide a valid assessment of CHNs’ practice competencies based on the Standards. However the results represent a self-assessment of CHNs’ learning needs which serves as a powerful tool for strengthening nursing undergraduate curricula and professional development.

Finally, slight revisions primarily related to reorganization of the CCHN standards and a few additions have occurred since the implementation of this study. Key changes include separating the first core standard into 3 standards to ensure equal weighting and importance to health promotion, prevention and health protection and health maintenance, restoration and palliation; greater emphasis of social determinants of health and social justice; primary focus on expected practices; and reordering the standards reflecting greater alignment to nursing practice. However, specific skills and knowledge expected of community health nurses have remained very much the same. Although data collection occurred in 2009, the competencies expected of community health nurses have remained much the same. Complimentary research currently being conducted in the public health sector in Canada is showing similar results in relation to areas requiring capacity building within the public health sector [42]. Therefore, it is felt that the results continue to hold relevance for Canadian CHNs.

## Conclusions

Due to fiscal constraints now recognized within Canada and elsewhere and the escalating costs of hospital-based curative care [43,44] decision makers within our health systems are more primed than ever before to acknowledge the value of health promotion, disease prevention. To ensure a strong nursing presence within the forefront of community health, it is imperative that decision-makers advocate for policies and allocation of resources to support the learning needs of CHNs to enable their continued response to the changing health needs of Canadians. The results of this study also need to be seriously considered by curriculum planners within schools of nursing to ensure a future nursing workforce prepared for the realignment of our health care system. In addition, CHN practice leaders and educators need to consider these results in determining where to strengthen content in professional development programs. For practicing CHNs, educational content needs to be tailored based on learner’s years of experience working in the community and their employment sector. Given ongoing curriculum revisions in undergraduate educational nursing programs, changing work contexts, as well as continued renewal of standards and competencies, future research should repeat such surveys to track changes in learning needs. Using the validated tool used in this study [20], modified to address any new competencies, will allow tracking of changes over time and help ensure that curriculum content is current and meeting CHNs’ learning needs in the future. Lastly, nurses need to take responsibility to identify and address their own learning needs through performance reviews.

## Abbreviations

CHNs: Community health nurses; CCHN Standards: Canadian Community Health Nursing Standards of Practice.

## Competing interests

The authors declare that they have no competing interests.

## Authors’ contributions

RV, RS, NA, AB, RMM, and JU participated in the conceptualization and design of the study. RV and RS oversaw all data collection. NA and SI performed the statistical analysis. All authors participated in the interpretation of results, helped to draft the manuscript, as well as read and approved the final manuscript.

## Pre-publication history

The pre-publication history for this paper can be accessed here:

http://www.biomedcentral.com/1472-6955/13/31/prepub

## Supplementary Material

Additional file 1 **Community Health Nurses’ Continuing Education Needs Questionnaire.** The addition file is the questionnaire that was used to conduct the survey. Click here for file
