# Supplementary material for: Community health nurses’ learning needs in relation to the Canadian community health nursing standards of practice: results from a Canadian survey
Source: BMC Nurs. 2014 Oct 21;13:31. doi: 10.1186/1472-6955-13-31 (PMC4209163; doi:10.1186/1472-6955-13-31)
Supplement: Additional file 1 — Community Health Nurses’ Continuing Education Needs Questionnaire. The addition file is the questionnaire that was used to conduct the survey. [file 1472-6955-13-31-S1.doc]

**
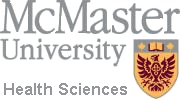
Community Health Nurses’**

**Continuing Education Needs Questionnaire**

| **Questionnaire Instructions:**   - Use only blue or black ballpoint pen; - Carefully read each statement below; - Base your responses on ONE nursing position that represents your community nursing practice at your current, ***primary*** workplace; - Choose your level of agreement with **each item** from each of the **two** rating scales,   as shown below:   - **Response Options: include both column 1 and column 2** - For example: - Choose *not applicable* (n/a) if the item does not apply to you or your workplace setting;  | **Activity Statement (Questionnaire Item)**  **Fill in circles or squares like this:● or ■; not like this: ⮿ ⦸.** | **I perform the stated activity:** | | | | | | | **I need more education related to this activity:** | | | | | | --- | --- | --- | --- | --- | --- | --- | --- | --- | --- | --- | --- | --- | |  | **Not applicable** | **Never** | **Rarely** | **Sometimes** | **Frequently** | **Always** | **Unsure** | **Completely disagree** | **Generally disagree** | **Neither agree nor disagree** | **Generally agree** | **Completely agree** | | 1. **In collaboration with the individual/community, I conduct assessments of:**    1. **individual *needs*.** | □ ○ ○ ○ ○ ● □ | | | | | | | ○ ○ ○ ○ ● | | | | |  - Choose *unsure* if you are undecided about the item; - Fill in circles or squares like this: ● or ■; not like this: ⮿ ⦸; - Complete the **Demographic Data Form** (following the questionnaire); - **Return** your completed questionnaire in the envelope provided. |
| --- | --- | --- | --- | --- | --- | --- | --- | --- | --- | --- | --- | --- | --- | --- | --- | --- | --- | --- | --- | --- | --- | --- | --- | --- | --- | --- | --- | --- | --- | --- | --- | --- | --- | --- | --- | --- | --- | --- | --- |

**Questionnaire begins on p. 2 👉**

**START HERE**

| **Activity Statement (Questionnaire Item)** | **I perform the stated activity:** | | | | | | | **I need more education related to this activity:** | | | | |
| --- | --- | --- | --- | --- | --- | --- | --- | --- | --- | --- | --- | --- |
| **Fill in circles or squares like this:● or ■; not like this: ⮿ ⦸.** | **Not applicable** | **Never** | **Rarely** | **Sometimes** | **Frequently** | **Always** | **Unsure** | **Completely disagree** | **Generally disagree** | **Neither agree nor disagree** | **Generally agree** | **Completely agree** |
| **STANDARD 1: PROMOTING HEALTH** |  | | | | | | |  | | | | |
| 1. **I use relevant information sources from multiple jurisdictional levels (e.g., local, regional, provincial/ territorial, and national).** | □ ○ ○ ○ ○ ○ □ | | | | | | | ○ ○ ○ ○ ○ | | | | |
| 1. **I use research findings.** | □ ○ ○ ○ ○ ○ □ | | | | | | | ○ ○ ○ ○ ○ | | | | |
| 1. **I use nursing informatics (i.e., information and communication technology) which includes generation, management, and processing of relevant data to support nursing practice.** | □ ○ ○ ○ ○ ○ □ | | | | | | | ○ ○ ○ ○ ○ | | | | |
| 1. **In collaboration with the individual/community, I conduct assessments of:** 2. **individual *needs*.** | □ ○ ○ ○ ○ ○ □ | | | | | | | ○ ○ ○ ○ ○ | | | | |
| 1. **individual *assets*, including available resources.** | □ ○ ○ ○ ○ ○ □ | | | | | | | ○ ○ ○ ○ ○ | | | | |
| 1. **community *needs*.** | □ ○ ○ ○ ○ ○ □ | | | | | | | ○ ○ ○ ○ ○ | | | | |
| 1. **community *assets*, including available resources.** | □ ○ ○ ○ ○ ○ □ | | | | | | | ○ ○ ○ ○ ○ | | | | |
| 1. **I address root causes of illness and disease.** | □ ○ ○ ○ ○ ○ □ | | | | | | | ○ ○ ○ ○ ○ | | | | |

| **Activity Statement (Questionnaire Item)** | **I perform the stated activity:** | | | | | | | **I need more education related to this activity:** | | | | |
| --- | --- | --- | --- | --- | --- | --- | --- | --- | --- | --- | --- | --- |
| **Fill in circles or squares like this:● or ■; not like this: ⮿ ⦸.** | **Not applicable** | **Never** | **Rarely** | **Sometimes** | **Frequently** | **Always** | **Unsure** | **Completely disagree** | **Generally disagree** | **Neither agree nor disagree** | **Generally agree** | **Completely agree** |
| 1. **I assist the individual/community to take responsibility for improving their health by increasing their knowledge of the determinants of health.** | □ ○ ○ ○ ○ ○ □ | | | | | | | ○ ○ ○ ○ ○ | | | | |
| 1. **I use social marketing strategies (i.e., media advocacy) to:** 2. **raise consciousness of health issues.** | □ ○ ○ ○ ○ ○ □ | | | | | | | ○ ○ ○ ○ ○ | | | | |
| 1. **shift social norms.** | □ ○ ○ ○ ○ ○ □ | | | | | | | ○ ○ ○ ○ ○ | | | | |
| 1. **In partnership with stakeholders, I evaluate population health promotion programs systematically.** | □ ○ ○ ○ ○ ○ □ | | | | | | | ○ ○ ○ ○ ○ | | | | |
| 1. **I select the appropriate level of preventative intervention [i.e., primary (immunization); secondary (screening); and tertiary (treatment and palliation)].** | □ ○ ○ ○ ○ ○ □ | | | | | | | ○ ○ ○ ○ ○ | | | | |
| 1. **I help individuals/communities:** 2. **make informed choices about protective health measures.** | □ ○ ○ ○ ○ ○ □ | | | | | | | ○ ○ ○ ○ ○ | | | | |
| 1. **make informed choices about preventative health measures.** | □ ○ ○ ○ ○ ○ □ | | | | | | | ○ ○ ○ ○ ○ | | | | |
| 1. **to identify potential risks to health.** | □ ○ ○ ○ ○ ○ □ | | | | | | | ○ ○ ○ ○ ○ | | | | |
| 1. **In a variety of contexts, including home, neighbourhood, workplace, school, and street, I utilize harm reduction principles to reduce risk factors.** | □ ○ ○ ○ ○ ○ □ | | | | | | | ○ ○ ○ ○ ○ | | | | |
| 1. **I engage in collaborative:** 2. **interdisciplinary partnerships to address prevention issues.** | □ ○ ○ ○ ○ ○ □ | | | | | | | ○ ○ ○ ○ ○ | | | | |
| 1. **intersectoral partnerships to address prevention issues.** | □ ○ ○ ○ ○ ○ □ | | | | | | | ○ ○ ○ ○ ○ | | | | |
| 13. **To ensure that the individual/ community receives effective service, I collaborate in using follow-up-systems.** | □ ○ ○ ○ ○ ○ □ | | | | | | | ○ ○ ○ ○ ○ | | | | |
| 1. **I evaluate collaborative practice (i.e., personal, team, and/or intersectoral) in achieving individual/community health outcomes.** | □ ○ ○ ○ ○ ○ □ | | | | | | | ○ ○ ○ ○ ○ | | | | |
| 1. **I assess the individual/family/ population’s:** 2. **health status within the context of their *environmental support*.** | □ ○ ○ ○ ○ ○ □ | | | | | | | ○ ○ ○ ○ ○ | | | | |
| 1. **health status within the context of their *social supports*.** | □ ○ ○ ○ ○ ○ □ | | | | | | | ○ ○ ○ ○ ○ | | | | |
| 1. **functional competence within the context of their *environmental support*.** | □ ○ ○ ○ ○ ○ □ | | | | | | | ○ ○ ○ ○ ○ | | | | |
| 1. **I develop a mutually agreed upon plan of care with the individual/ family.** | □ ○ ○ ○ ○ ○ □ | | | | | | | ○ ○ ○ ○ ○ | | | | |
| 1. **I identify a range of interventions, including health promotion, disease prevention, and direct clinical care strategies (including those related to palliation).** | □ ○ ○ ○ ○ ○ □ | | | | | | | ○ ○ ○ ○ ○ | | | | |
| 1. **I maximize the ability of an individual/family/community to take responsibility for their health needs according to available resources.** | □ ○ ○ ○ ○ ○ □ | | | | | | | ○ ○ ○ ○ ○ | | | | |

| **Activity Statement (Questionnaire Item)** | **I perform the stated activity:** | | | | | | | **I need more education related to this activity:** | | | | |
| --- | --- | --- | --- | --- | --- | --- | --- | --- | --- | --- | --- | --- |
| **Fill in circles or squares like this:● or ■; not like this: ⮿ ⦸.** | **Not applicable** | **Never** | **Rarely** | **Sometimes** | **Frequently** | **Always** | **Unsure** | **Completely disagree** | **Generally disagree** | **Neither agree nor disagree** | **Generally agree** | **Completely agree** |
| 1. **I support informed choice of the individual/family/community’s specific requests while recognizing their:** 2. **diversity.** | □ ○ ○ ○ ○ ○ □ | | | | | | | ○ ○ ○ ○ ○ | | | | |
| 1. **abilities.** | □ ○ ○ ○ ○ ○ □ | | | | | | | ○ ○ ○ ○ ○ | | | | |
| 1. **I respect the individual/family/ community’s specific requests while recognizing their abilities.** | □ ○ ○ ○ ○ ○ □ | | | | | | | ○ ○ ○ ○ ○ | | | | |
| 1. **I adapt community health nursing techniques/approaches/procedures to the challenges inherent to the particular community situation/setting.** | □ ○ ○ ○ ○ ○ □ | | | | | | | ○ ○ ○ ○ ○ | | | | |
| 1. **I apply epidemiological principles in using strategies (such as, a) screening, b) surveillance, c) communicable disease response, d) outbreak management, and e) education).** | □ ○ ○ ○ ○ ○ □ | | | | | | | ○ ○ ○ ○ ○ | | | | |
| 1. **I recognize trends in epidemiological data.** | □ ○ ○ ○ ○ ○ □ | | | | | | | ○ ○ ○ ○ ○ | | | | |
| 1. **I facilitate maintenance of health in response to significant emergencies that negatively impact upon the health of clients.** | □ ○ ○ ○ ○ ○ □ | | | | | | | ○ ○ ○ ○ ○ | | | | |
| **STANDARD 2: BUILDING INDIVIDUAL/COMMUNITY CAPACITY** |  | | | | | | |  | | | | |
| 1. **I use community development principles when I:** 2. **engage the individual/community in a consultative process.** | □ ○ ○ ○ ○ ○ □ | | | | | | | ○ ○ ○ ○ ○ | | | | |
| 1. **use empowering strategies (such as mutual goal setting, visioning, and facilitation).** | □ ○ ○ ○ ○ ○ □ | | | | | | | ○ ○ ○ ○ ○ | | | | |
| 1. **use facilitation skills to support group development.** | □ ○ ○ ○ ○ ○ □ | | | | | | | ○ ○ ○ ○ ○ | | | | |
| 1. **assist the group/community to marshal available resources to support taking action on their health issues.** | □ ○ ○ ○ ○ ○ □ | | | | | | | ○ ○ ○ ○ ○ | | | | |
| 1. **I use a comprehensive mix of community/population based strategies (such as coalition building, intersectoral partnerships, and networking) to address issues of concern to groups/populations.** | □ ○ ○ ○ ○ ○ □ | | | | | | | ○ ○ ○ ○ ○ | | | | |
| 1. **I support the individual/family/ community/population in developing skills of self-advocacy.** | □ ○ ○ ○ ○ ○ □ | | | | | | | ○ ○ ○ ○ ○ | | | | |
| 1. **I use principles of social justice to support those who are unable to take action for themselves.** | □ ○ ○ ○ ○ ○ □ | | | | | | | ○ ○ ○ ○ ○ | | | | |
| **STANDARD 3: BUILDING RELATIONSHIPS** |  | | | | | | |  | | | | |
| 1. **I recognize my own personal perspective (such as attitudes, beliefs, assumptions, feelings, and values) about their potential effect on interventions with individuals/communities.** | □ ○ ○ ○ ○ ○ □ | | | | | | | ○ ○ ○ ○ ○ | | | | |
| 1. **I identify the individual/ community’s perspective (such as beliefs, attitudes, feelings, and values) about health.** | □ ○ ○ ○ ○ ○ □ | | | | | | | ○ ○ ○ ○ ○ | | | | |
| 1. **I am aware of culturally relevant communication in building relationships.** | □ ○ ○ ○ ○ ○ □ | | | | | | | ○ ○ ○ ○ ○ | | | | |
| 1. **I provide culturally relevant care in diverse communities.** | □ ○ ○ ○ ○ ○ □ | | | | | | | ○ ○ ○ ○ ○ | | | | |
| 1. **I trust and respect the individual/ family/community’s ability to:** 2. **identify their health issues.** | □ ○ ○ ○ ○ ○ □ | | | | | | | ○ ○ ○ ○ ○ | | | | |
| 1. **solve their own problems.** | □ ○ ○ ○ ○ ○ □ | | | | | | | ○ ○ ○ ○ ○ | | | | |
| 1. **I maintain awareness of community resources.** | □ ○ ○ ○ ○ ○ □ | | | | | | | ○ ○ ○ ○ ○ | | | | |
| 1. **I negotiate an end to the individual/ family /community relationship.** | □ ○ ○ ○ ○ ○ □ | | | | | | | ○ ○ ○ ○ ○ | | | | |
| **STANDARD 4: FACILITATING**  **ACCESS AND EQUITY** |  | | | | | | |  | | | | |
| 1. **I assess individual and community capacities (such as norms, values, beliefs, knowledge, resources, and power structures).** | □ ○ ○ ○ ○ ○ □ | | | | | | | ○ ○ ○ ○ ○ | | | | |
| 1. **I support individuals/communities in their choice to access alternate health care options.** | □ ○ ○ ○ ○ ○ □ | | | | | | | ○ ○ ○ ○ ○ | | | | |
| 1. **I refer to services within:** 2. **the *health* sector.** | □ ○ ○ ○ ○ ○ □ | | | | | | | ○ ○ ○ ○ ○ | | | | |
| 1. ***other* sectors.** | □ ○ ○ ○ ○ ○ □ | | | | | | | ○ ○ ○ ○ ○ | | | | |
| 1. **I coordinate access to services within:** 2. **the *health* sector.** | □ ○ ○ ○ ○ ○ □ | | | | | | | ○ ○ ○ ○ ○ | | | | |
| 1. ***other* sectors.** | □ ○ ○ ○ ○ ○ □ | | | | | | | ○ ○ ○ ○ ○ | | | | |
| 1. **I provide programs to individuals/ communities using delivery methods that are responsive to their needs.** | □ ○ ○ ○ ○ ○ □ | | | | | | | ○ ○ ○ ○ ○ | | | | |
| 1. **I use strategies (such as home visits, outreach, and case finding) for vulnerable populations to ensure access to services.** | □ ○ ○ ○ ○ ○ □ | | | | | | | ○ ○ ○ ○ ○ | | | | |
| 1. **To address service accessibility issues, I take action, based on evidence, with individuals/ communities at the:** 2. **organizational.** | □ ○ ○ ○ ○ ○ □ | | | | | | | ○ ○ ○ ○ ○ | | | | |
| 1. **municipal.** | □ ○ ○ ○ ○ ○ □ | | | | | | | ○ ○ ○ ○ ○ | | | | |
| 1. **provincial/territorial.** | □ ○ ○ ○ ○ ○ □ | | | | | | | ○ ○ ○ ○ ○ | | | | |
| 1. **federal levels.** | □ ○ ○ ○ ○ ○ □ | | | | | | | ○ ○ ○ ○ ○ | | | | |
| **STANDARD 5: DEMONSTRATING PROFESSIONAL RESPONSIBILITY AND ACCOUNTABILITY** |  | | | | | | |  | | | | |
| 1. **I take preventive action to protect individuals/communities from:** 2. **unsafe circumstances.** | □ ○ ○ ○ ○ ○ □ | | | | | | | ○ ○ ○ ○ ○ | | | | |
| 1. **unethical circumstances.** | □ ○ ○ ○ ○ ○ □ | | | | | | | ○ ○ ○ ○ ○ | | | | |
| 1. **I take action on factors which impinge on:** 2. **autonomy of practice.** | □ ○ ○ ○ ○ ○ □ | | | | | | | ○ ○ ○ ○ ○ | | | | |
| 1. **quality of care.** | □ ○ ○ ○ ○ ○ □ | | | | | | | ○ ○ ○ ○ ○ | | | | |
| 1. **I participate in the advancement of community health nursing by:** 2. **mentoring students.** | □ ○ ○ ○ ○ ○ □ | | | | | | | ○ ○ ○ ○ ○ | | | | |
| 1. **mentoring novice practitioners.** | □ ○ ○ ○ ○ ○ □ | | | | | | | ○ ○ ○ ○ ○ | | | | |
| 1. **I participate in professional activities.** | □ ○ ○ ○ ○ ○ □ | | | | | | | ○ ○ ○ ○ ○ | | | | |
| 1. **I seek professional development experiences that are consistent with current community health nursing practice.** | □ ○ ○ ○ ○ ○ □ | | | | | | | ○ ○ ○ ○ ○ | | | | |
| 1. **I address nursing issues that will affect the population through:** 2. **personal advocacy.** | □ ○ ○ ○ ○ ○ □ | | | | | | | ○ ○ ○ ○ ○ | | | | |
| 1. **participation in relevant professional associations.** | □ ○ ○ ○ ○ ○ □ | | | | | | | ○ ○ ○ ○ ○ | | | | |
| 1. **I make decisions using ethical standards/principles, taking into consideration the tension between individual versus the societal good of all people.** | □ ○ ○ ○ ○ ○ □ | | | | | | | ○ ○ ○ ○ ○ | | | | |
| 1. **I seek assistance with problem solving, as needed, to determine the best course of action in response to:** 2. **ethical dilemmas.** | □ ○ ○ ○ ○ ○ □ | | | | | | | ○ ○ ○ ○ ○ | | | | |
| 1. **new situations.** | □ ○ ○ ○ ○ ○ □ | | | | | | | ○ ○ ○ ○ ○ | | | | |
| 1. **I address nursing issues that will affect the population through personal advocacy.** | □ ○ ○ ○ ○ ○ □ | | | | | | | ○ ○ ○ ○ ○ | | | | |
| 1. **I contribute proactively to the quality of the work environment by:** 2. **identifying solutions.** | □ ○ ○ ○ ○ ○ □ | | | | | | | ○ ○ ○ ○ ○ | | | | |
| 1. **mobilizing colleagues.** | □ ○ ○ ○ ○ ○ □ | | | | | | | ○ ○ ○ ○ ○ | | | | |
| 1. **actively participating in team/organizational structures.** | □ ○ ○ ○ ○ ○ □ | | | | | | | ○ ○ ○ ○ ○ | | | | |

| **Activity Statement (Questionnaire Item)** | **I perform the stated activity:** | | | | | | | **I need more education related to this activity:** | | | | |
| --- | --- | --- | --- | --- | --- | --- | --- | --- | --- | --- | --- | --- |
| **Fill in circles or squares like this:● or ■; not like this: ⮿ ⦸.** | **Not applicable** | **Never** | **Rarely** | **Sometimes** | **Frequently** | **Always** | **Unsure** | **Completely disagree** | **Generally disagree** | **Neither agree nor disagree** | **Generally agree** | **Completely agree** |
| 1. **I provide constructive feedback to peers to enhance community health nursing practice.** | □ ○ ○ ○ ○ ○ □ | | | | | | | ○ ○ ○ ○ ○ | | | | |
| 1. **I document community health nursing activities, including telephone advice and work with clients, in a thorough manner.** | □ ○ ○ ○ ○ ○ □ | | | | | | | ○ ○ ○ ○ ○ | | | | |
| 1. **I advocate for:** 2. **effective/efficient use of community health nurse resources.** | □ ○ ○ ○ ○ ○ □ | | | | | | | ○ ○ ○ ○ ○ | | | | |
| 1. **resource allocation for individuals, groups, and populations, to facilitate access to conditions for health and health services.** | □ ○ ○ ○ ○ ○ □ | | | | | | | ○ ○ ○ ○ ○ | | | | |
| 1. **healthy public policy, by participating in legislative and policymaking activities that influence health determinants.** | □ ○ ○ ○ ○ ○ □ | | | | | | | ○ ○ ○ ○ ○ | | | | |
| 1. **I use reflective practice as a means of continually seeking to improve personal community health nursing practice.** | □ ○ ○ ○ ○ ○ □ | | | | | | | ○ ○ ○ ○ ○ | | | | |
| 1. **I use available resources to systematically evaluate community health nursing practice (e.g., availability, acceptability, quality, efficiency, and effectiveness).** | □ ○ ○ ○ ○ ○ □ | | | | | | | ○ ○ ○ ○ ○ | | | | |

You are almost done!

The last few items relate to the theoretical frameworks underpinning the Canadian Community Health Nursing Standards of Practice: the Population Health Promotion Model, the Ottawa Charter models, and the Jakarta Declaration. **Not all community health nurses may be familiar** **with these documents.** If you are not familiar with this content:

- Mark ■ **unsure** in the first column (**I perform the stated activity**) following the Activity Statements.
- Then, complete the second column (**I need more education related to this activity**) following the Activity Statements to indicate your need for education about this activity.

| **Activity Statement (Questionnaire Item)** | **I perform the stated activity:** | | | | | | | **I need more education related to this activity:** | | | | |
| --- | --- | --- | --- | --- | --- | --- | --- | --- | --- | --- | --- | --- |
| **Fill in circles or squares like this:● or ■; not like this: ⮿ ⦸.** | **Not applicable** | **Never** | **Rarely** | **Sometimes** | **Frequently** | **Always** | **Unsure** | **Completely disagree** | **Generally disagree** | **Neither agree nor disagree** | **Generally agree** | **Completely agree** |
| 1. **I facilitate planned change through applying the Population Health Promotion Model.** | □ ○ ○ ○ ○ ○ □ | | | | | | | ○ ○ ○ ○ ○ | | | | |
| 1. **I implement health promotion strategies based on the Ottawa Charter.** | □ ○ ○ ○ ○ ○ □ | | | | | | | ○ ○ ○ ○ ○ | | | | |
| 1. **I facilitate action in support of the *five* priorities of the Jakarta Declaration.** | □ ○ ○ ○ ○ ○ □ | | | | | | | ○ ○ ○ ○ ○ | | | | |

**THANK YOU for your contribution to this study.**

**Please continue to the Demographic Data Form.**

**Demographic Data Form**

**Instructions**: When answering questions, fill in circles like this: ; not like this:  .

| **1. Your *title* in nursing.** | RN* RN (Extended Class) Nurse Practitioner    ○ ○ ○ |
| --- | --- |
| **2. *Highest* level of education in nursing.** | Certificate Diploma Degree Masters Doctorate  ○ ○ ○ ○ ○ |
| **3. Highest level of education in *other* than nursing.** | n/a* Diploma Degree Masters Doctorate  ○ ○ ○ ○ ○ |
| **4. Number of years in nursing in *total*.** | Under 2yrs 2-5 yrs 6-10yrs 11-15yrs 16-20yrs 21-25yrs 25+yrs    ○ ○ ○ ○ ○ ○ ○ |
| **5. Number of years nursing in the *community*.** | Under 2yrs 2-5 yrs 6-10yrs 11-15yrs 16-20yrs 21-25yrs 25+yrs    ○ ○ ○ ○ ○ ○ ○ |
| **6. I am employed at my *Primary* Employer:**  **Employer 2**  **Employer 3** | Full-Time Part-Time Casual Contract  ○ ○ ○ ○  ○ ○ ○ ○  ○ ○ ○ ○ |

*RN=Registered Nurse, n/a=not applicable

**CURRENT** Employment Information: Answer the following questions relative to your ***Primary*** *Employer*.

| **7. Place of work:**  **Choose only ONE based on your *primary* employer.** | ○ Public Health Unit/Department  ○ Community Health Agency  ○ Community Nursing Clinic  ○ Mental Health Centre  ○ Community Health Centre  ○ Physician’s Office/Family Practice Unit  ○ Home Health  ○ Extra-Mural Program (NB only)  ○ Private Nursing Agency  ○ Visiting Nursing Agency  ○ CCAC (ON only)  ○ First Nations and Inuit Health Branch  ○ Indian Reserve employed  ○ Outposts/Nursing Stations  ○ Association/Government  ○ Business/Industry/Occupational Health  ○ Educational Institution  ○ Parish Nursing  ○ Armed Forces  ○ Addiction Centre  ○ Self-employed/Independent Practice  ○ Dentist’s office  ○ Other Community (please specify): _______________________ |
| --- | --- |
| **8. Position:**  **Choose only ONE based on your *primary* employer.** | ○ Chief Nursing Officer/CEO/Director/Assistant Director  ○ Clinical Nurse Specialist  ○ Clinical Resources Nurse/Clinical Educator  ○ Consultant  ○ Coordinator/Case Manager  ○ Home Health Nurse  ○ Instructor/Professor/Educator  ○ Manager/Supervisor/Administrator  ○ Nurse Practitioner/Extended Practice  ○ Occupational Health Nurse  ○ Outpost Nurse  ○ Public Health Nurse  ○ Mental Health Nurse/Registered Psychiatric Nurse  ○ Researcher, Policy or Informatics Analyst  ○ Staff Nurse  ○ Family Practice Nurse  ○ Visiting Nurse  ○ Other (please specify):_________________________________ |
| **9. Jurisdiction in which you *currently* work:**  **Choose only ONE based on your *primary* employer.** | ○ British Columbia  ○ Alberta  ○ Saskatchewan  ○ Manitoba  ○ Ontario  ○ Quebec  ○ New Brunswick  ○ Prince Edward Island  ○ Nova Scotia  ○ Newfoundland and Labrador  ○ Yukon Territory  ○ Northwest Territories  ○ Nunavut  ○ Other (Please specify):_________________________________ |
| **10. Gender** | ○ Male ○ Female |
| **11. *Year* of birth** |  |

**THANK YOU for your participation!**

Please **return** all study materials **today** in the enclosed stamped, addressed envelope to:

Danielle Hunter

McMaster University

1200 Main Street West, HSC 3N28

Hamilton, ON   L8N 3Z5

Tel:  (905) 525-9140, ext. 21222

by Fax: (905) 521-8834 or by email: dhunte@mcmaster.ca
